# Lymphoepithelial carcinoma: a case report of a rare tumor of the larynx

**DOI:** 10.1186/s12907-017-0061-0

**Published:** 2017-11-25

**Authors:** Nawal Hammas, Najib Benmansour, Mohamed Nour-dine El Alami El Amine, Laila Chbani, Hind El Fatemi

**Affiliations:** 1 Department of Pathology, Hassan II University Hospital, 30000 Fez, Morocco; 2 Biomedical and Translational Research Laboratory, Faculty of Medicine and Pharmacy, Sidi Mohamed Ben Abdellah University, Fez, Morocco; 3 Department of otorhinolaryngology, HASSAN II University Hospital, Faculty of Medicine and Pharmacy, Sidi Mohammed Ben Abdellah University, Fez, Morocco

**Keywords:** Larynx, Lymphoepithelial carcinoma, Epstein- Barr virus

## Abstract

**Background:**

Lymphoepithelial carcinoma is a tumor mostly diagnosed in the nasopharynx, but it has also been described in a variety of nonnasopharyngeal sites. It is extremely rare in the larynx and should be distinguished from squamous cell carcinoma. Therefore, it must be known by clinicians, pathologists and oncologists. In this case report, we discuss its etiopathogeny, its epidemiological, clinical, pathological and therapeutic aspects, and its outcome.

**Case presentation:**

An 81-year-old Morrocan man, smoker for 40 years, presented with a 1 year history of dysphonia, dyspnea and dysphagia. Laryngoscopy showed a mass occupying supraglottic, glottic and subglottic levels of the larynx. Cervico-thoracic computed tomography scan showed a laryngeal wall thickening with cervical lymphadenopathy. Laryngeal biopsy was performed. Microscopic analysis and immunohistochemistry confirmed the diagnosis of laryngeal lymphoepithelial carcinoma. Immunostaining for LMP1 was negative.

**Conclusion:**

Laryngeal lymphoepithelial carcinoma is an extremely rare and an aggressive tumor. It is rarely associated with the EBV. It must be regarded as a distinct entity. Radiotherapy is advisable as the unique therapy for local tumor. A correct diagnosis and a close collaboration between the pathologist and clinicians is mandatory for an optimal treatment strategy.

## Background

Lymphoepithelial carcinoma (LEC) is a neoplasm mostly located in the nasopharynx where it represents 40% of all neoplasms [1, 2]. It occurs worldwide but it has an endemic geographic distribution, particularly in Southeast Asia and Eskimos [3]. Nonnasopharyngeal LEC has also been reported in other locations, such as sinonasal tract, nasolacrimal duct, oral cavity, oropharynx, salivary glands, thymus, hypopharynx, esophagus, stomach, trachea, lung, and others [1–4]. Many terms have been used for nonnasopharyngeal LEC like undifferentiated carcinoma of nasopharyngeal type, undifferentiated carcinoma with lymphoid stroma, lymphoepithelioma, lymphoepithelial-like carcinoma and lymphoepithelial carcinoma. The latter has been approved by the World Health Organization [5]. Nonnasopharyngeal and nasopharyngeal LEC have the same microscopic appearance but their relationship to EBV is different. In fact, the former is less likely associated to EBV [2, 3].

LEC is extremely rare in the larynx [4]. We present herein one new laryngeal presentation and, through this case, we discuss the clinical and histopathologic findings, the diagnosis problems and therapeutic aspects of this rare neoplasm.

## Case presentation

**Fig. 1:**
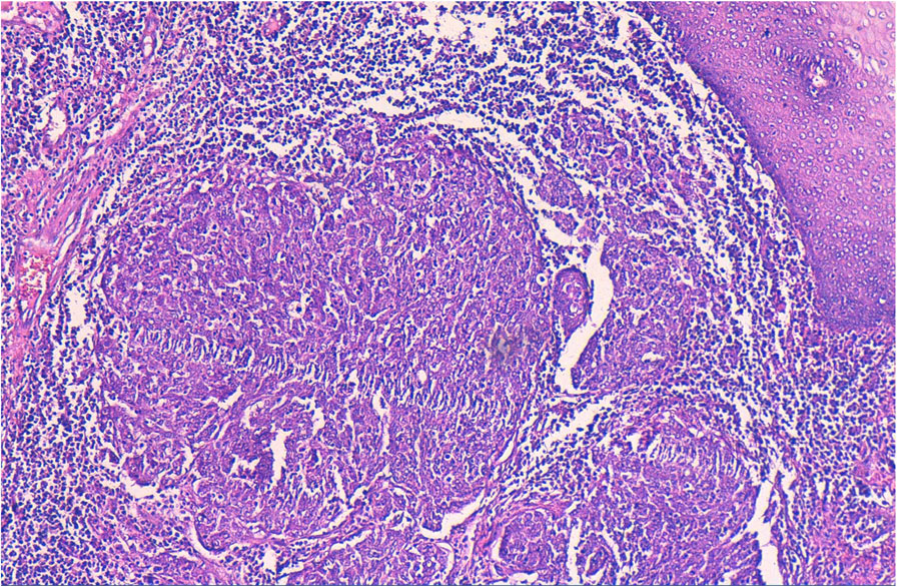
Microscopic appearance: the tumor is composed of compact nests and sheets of epithelial cells surrounded by a prominent component of mature lymphocytes and plasma cells. Hematoxylin and eosin stain; original magnification ×100

**Fig. 2:**
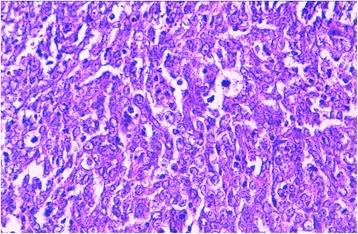
Microscopic appearance: tumor cells are undifferentiated, large, with round, vesicular nuclei, containing a prominent nucleolus, and with an abundant, ill defined cytoplasm. Hematoxylin and eosin stain; original magnification ×400

**Fig. 3:**
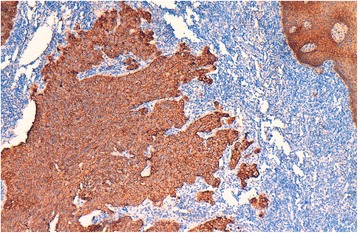
positive immunohistochemical staining for cytokeratin 5/6

An 81-year-old Morrocan man, smoker for 40 years, presented with a 1 year history of dysphonia, dyspnea and dysphagia. Laryngoscopy showed a mass occupying supraglottic, glottic and subglottic levels of the larynx, with extension to the epiglottis, the tongue base and the retro-cricoid area. Cervico-thoracic computed tomography scan showed a laryngeal wall thickening especially at the glottic and supraglottic levels with cervical lymphadenopathy without distant metastasis. Laryngeal biopsy was performed and revealed, on microscopic examination, a malignant tumor composed of solid sheets disposed in lymphoid background (Fig. 1). Tumor cells were round, large, poorly differentiated, nonkeratinized, and contained large round vesicular nuclei with prominent nucleoli. The cytoplasm was poorly limited (Fig. 2). Surface epithelium exhibits some alterations like hyperplasie and keratosis, without dysplasia. Automated immunohistochemistry showed positivity for Cytokeratin 5/6 (D5/16B4) (Fig. 3). Neuroendocrine markers (chromogranin and synaptophysin), melanoma markers (Melan A and HMB45), myogenic markers (desmin and smooth muscle actin), LCA (leucocyt commun antigen), CD99 and CD117 were negative. These histological and immunohistochemical results confirmed the diagnosis of laryngeal lymphoepithelial carcinoma. Screening for EBV by immunohistochemistry using anti-LMP 1 antibody (latent membrane protein 1) was negative. The patient underwent tracheotomy and radiotherapy.

## Discussion

LEC of the larynx is an extremely rare and aggressive tumor which accounts for 0.2% of all laryngeal cancers [2, 4, 6]. It most commonly originates from supraglottic region, usually centered around the ventricles or involving the epiglottis [2, 3, 6]. It occurs mostly in older adults (mean 62 years) with male predominance (male/female ratio = 3:1) [3]. The main symptom is dysphagia or hoarseness [7]. In our case, the tumor was occupying all larynx and was accompanied by cervical lymphadenopathy.

Macroscopically, the tumor forms a mass that may be ulcerated [5]. Diagnosis can be challenging because the tumor may arise from hidden, submucosal sites. Microscopically, laryngeal LEC is similar to the nasopharyngeal one. It comprises solid sheets or irregular islands of malignant epithelial cells intimately intermingled with prominent component of lymphocytes and plasma cells [2–4]. Tumor cells are large, with indistinct cell borders, round to oval vesicular nuclei, and a single large central nucleoli [3]. A component of squamous cell carcinoma accounting for 10-75% of the tumour may be seen in about half of the cases. This component was not found in our case. The overlying epithelium can show carcinoma-in-situ [5]. Immunohistochemically, tumor cells show positive staining for keratin and epithelial membrane antigen [2]. In the present case, immunohistochemical stains for cytokeratin was positive and constituted a proof of epithelial differentiation.

Distinction between LEC on the one hand and large cell lymphoma and melanoma on the other hand is pronostically important and can at times be difficult. Immunohistochemistry is essential in differential diagnosis by demonstrating expression of cytokeratin in LEC. Immunostaining with melanocyte differentiation markers (HMB45 or Melan-A) and lymphoid markers is useful to eliminate melanoma and lymphoma respectively. Nasopharyngeal carcinoma with laryngeal metastasis must also be eliminated [5, 8, 9].

The relationship between EBV and LEC of the larynx remains controversial. Laryngeal LEC is less likely associated with EBV than its nasopharyngeal conterpart [2, 6]. MacMillan et al. [3] studied eight cases of LEC of the larynx and hypopharynx. They found that none of the cases was positive for EBV and suggested that the EBV has a limited role in the etiopathogenesis of this tumor in patients of non-Asian descent. The same conclusion was proposed by Marioni et al. [10] who found, among 16 cases evaluated for the presence of EBV, only four cases (25%) positive for this virus. In our case, immunostaining for EBV was negative.

In order to evaluate the modes of invasion of laryngeal and pharyngeal carcinomas, Micheau et al. [11] studied 2430 laryngectomy and pharyngolaryngectomy surgical specimens. They found a single or double laryngocele in 70% of the cases. Histolologic analysis of the laryngoceles showed cylindrical or squamous epithelium with organized lymphoid tissue, similar of the histology of lymph nodes and lymphoid structures of Waldeyer’s ring. This lymphoid-lined structure is probably the site of origin of laryngeal LEC. Alternatively, Toker and Peterson [12] postulated that the site of origine of these lesions may be active basal epithelium of the larynx, which is similar to epithelium of tonsillar crypts. Relationship between LEC and smoking is different in laryngeal and nasopharyngeal location. In the former, smoking may play a role while in the latter, it is not considered to be a risk factor [1, 4]. In our case, the patient had a long history of smoking.

Laryngeal LEC is a highly radiosensitive disease and radiotherapy should be considered as the main treatment because it provides excellent local control rates [2, 4, 6]. The value of chemotherapy is still unknown. Neoadjuvant chemotherapy may be recommanded in cases of early regional adenopathy with the aim of decreasing the distant metastasis rate [4, 6].

Laryngeal LEC shares many characteristics with its nasopharyngeal counterpart. They both have significant susceptibility for early regional and distant metastases. The initial stage is the primary determinant of prognosis. Death from disease occurs in about one third of patients. [3, 4, 13].

## Conclusion

LEC of the larynx is an extremely rare and an aggressive tumor. It has the same microscopic features as its nasopharyngeal counterpart. Radiotherapy is advisable as the unique therapy for local tumor. A correct diagnosis and a close collaboration between the pathologist and clinicians is mandatory for an optimal treatment strategy.

## Abbreviations

EBV: Epstein-Barr Virus
LEC: Lymphoepithelial carcinoma
LMP 1: Latent membrane protein 1
